# OXCT1 Enhances Gemcitabine Resistance Through NF-κB Pathway in Pancreatic Ductal Adenocarcinoma

**DOI:** 10.3389/fonc.2021.698302

**Published:** 2021-11-05

**Authors:** Jinsheng Ding, Hui Li, Yang Liu, Yongjie Xie, Jie Yu, Huizhi Sun, Di Xiao, Yizhang Zhou, Li Bao, Hongwei Wang, Chuntao Gao

**Affiliations:** ^1^Department of Pancreatic Cancer, Key Laboratory of Cancer Prevention and Therapy, Tianjin Medical University Cancer Institute and Hospital, National Clinical Research Center for Cancer, Tianjin’s Clinical Research Center for Cancer, Tianjin, China; ^2^The Graduate School, Tianjin Medical University, Tianjin, China; ^3^Key Laboratory of Cancer Prevention and Therapy, Key Laboratory of Breast Cancer Prevention and Therapy, Tianjin Medical University Cancer Institute and Hospital, National Clinical Research Center for Cancer, Tianjin’s Clinical Research Center for Cancer, Tianjin, China

**Keywords:** Pancreatic ductal adenocarcinoma, OXCT1, gemcitabine, chemoresistance, NF-κB

## Abstract

**Background:**

Pancreatic ductal adenocarcinoma (PDAC) is a type of malignant tumor with a five-year survival rate of less than 10%. Gemcitabine (GEM) is the most commonly used drug for PDAC chemotherapy. However, a vast majority of patients with PDAC develop resistance after GEM treatment.

**Methods:**

We screened for GEM resistance genes through bioinformatics analysis. We used immunohistochemistry to analyze 3-oxoacid CoA-transferase 1 (OXCT1) expression in PDAC tissues. The survival data were analyzed using the Kaplan–Meier curve. The expression levels of the genes related to OXCT1 and the NF-κB signaling pathway were quantified using real−time quantitative PCR and western blot analyses. We performed flow cytometry to detect the apoptosis rate. Colony formation assay was performed to measure the cell proliferation levels. The cytotoxicity assays of cells were conducted using RTCA. The downstream pathway of OXCT1 was identified *via* the Gene Set Enrichment Analysis. Tumor growth response to GEM *in vivo* was also determined in mouse models.

**Results:**

Bioinformatics analysis revealed that OXCT1 is the key gene leading to GEM resistance. Patients with high OXCT1 expression exhibited short relapse-free survival under GEM treatment. OXCT1 overexpression in PDAC cell lines exerted inhibitory effect on apoptosis after GEM treatment. However, the down-regulation of OXCT1 showed the opposite effect. Blocking the NF-κB signaling pathway also reduced GEM resistance of PDAC cells. Tumor growth inhibition induced by GEM *in vivo* reduced after OXCT1 overexpression. Moreover, the effect of OXCT1 on GEM refractoriness in PDAC cell lines was reversed through using an NF-κB inhibitor.

**Conclusion:**

OXCT1 promoted GEM resistance in PDAC *via* the NF-κB signaling pathway both *in vivo* and *in vitro*. Our results suggest that OXCT1 could be used as a potential therapeutic target for patients with PDAC.

## Introduction

Pancreatic ductal adenocarcinoma (PDAC) is the most common type of pancreatic cancer. It is a highly malignant tumor with poor prognosis and a five-year survival rate of only 10% (1). It is projected to be the second leading cause of cancer-related deaths in the western world by 2030 (2–5). In recent years, the incidence of pancreatic cancer has been increasing, whereas the progress in its treatment has been rather slow. In 2014, 46,420 patients were newly diagnosed with pancreatic cancer, and 39,590 patients died from pancreatic cancer in the United States. In China, the mortalities attributed to pancreatic cancer ranks fifth among all cancer mortalities. Surgical operation is the most effective treatment for pancreatic cancer, and radical resection has been shown to considerably improve the survival rate of patients (6). However, only 10–15% of patients with PDAC have a chance to receive radical surgery clinically because most patients with PDAC are already at an advanced stage during diagnosis given that the early symptoms of pancreatic cancer are not noticeable (7–10). Therefore, at present, chemotherapy is an important means for the treatment of advanced PDAC (11). However, considerable drug resistance against gemcitabine (GEM), which is used as the first-line treatment for PDAC (12), develops after a certain period of its use. This gives rise to great challenges for PDAC treatment.

GEM is a deoxycytidine analog with multiple modes of action inside cells (13). It has been used as a classic chemotherapy agent for nearly 20 years (14). During this period, GEM has become the standard first-line treatment for advanced PDAC. The poor efficacy of GEM in the treatment of PDAC may be due to the difficulty of drug penetration into the dense and vascularized stroma of tumors (15). Many different cellular pathways, transcription factors, and nucleotide metabolic enzymes have been found to be associated with GEM resistance and sensitivity (16–19). Although GEM is used relatively widely and commonly, the mechanisms associated with GEM resistance remain unclear. Elucidating the relevant mechanisms of GEM resistance in PDAC is necessary to ultimately improve the survival rate of patients with PDAC.

In response to stress, stimuli, and malnutrition, cancer cells dysregulate some metabolic pathways and activate certain signaling pathways (20–22). These changes may cause metabolic disorders, eventually leading to cell damage and even death. Cells resist these adverse conditions through a series of compensatory mechanisms to escape from apoptosis and death. In 2016, Huang et al. confirmed that when the energy supply of cancer cells is insufficient, ketone metabolism is significantly enhanced to maintain the cellular needs (23). 3-Oxoacid CoA-transferase 1 (OXCT1) is a key rate-limiting enzyme in ketone body metabolism. The product of OXCT1 is converted into acetyl-CoA, which finally participates in the tricarboxylic acid cycle for oxidation and ATP production (24).

In this study, we first screened out the relevant target gene OXCT1 through bioinformatic analysis to explore the drug resistance mechanism of PDAC against GEM. Based on this analysis, we found that OXCT1 was highly expressed in tumor tissues and that its expression level was significantly negatively correlated with relapse-free survival (RFS). Through experimental validation, we found that OXCT1 could enhance GEM resistance in PDAC. Next, we identified that the NF-κB signaling pathway was a potential downstream pathway of OXCT1 and tested our hypothesis by inhibiting NF-κB signaling pathway. Our study provides new theoretical and experimental support for GEM tolerance in PDAC and identify a new potential therapeutic target.

## Material and Methods

### Patients and Samples in Public Database

The gene expression profiles of GSE12945 and GSE80617 were downloaded from the Gene Expression Omnibus (GEO) database (http://www.ncbi.nlm.nih.gov/geo/). The mRNA profiles of pancreatic tumors and adjacent normal tissues were generated *via* high-throughput sequencing based on GPL11154 Illumina HiSeq2000 (*Homo sapiens*). Twenty samples were obtained from 20 patients, among which the samples were paired consisting of tumor/normal specimens from the same patients. The gene expression profiles of tumor specimens were compared with those of normal specimens to identify the differentially expressed genes (DEGs). The Venn diagram of the DEGs was generated using three datasets. From these three datasets, 18 common co-expressed genes were identified. OXCT1 was selected for further analysis among all the identified DEGs.

### Identification of Differentially Expressed mRNAs

Log fold change (FC) and *P* value were used to filter the differentially expressed mRNAs among the three datasets. We selected the DEGs in accordance with the *P* value threshold and absolute value of FC. *P*<0.05 was considered to indicate statistically significant difference.

### Mice, Cell Culture, and Reagents

Female nude BALB/c mice, 6–8 weeks old, were obtained from SiPeiFu, Beijing, China. BxPC-3, MIA PaCa-2, AsPC-1, CFPAC-1, PANC-1, Pan02, SW1990, HPDE6C7, and 293T cell lines were used in this study. These cell lines were obtained from the American Type Culture Collection. We used DMEM or RPMI-1640 (Gibco) medium supplemented with 10% fetal bovine serum (Gibco) to culture the cells in a humidified 5% CO_2_ atmosphere at 37°C. GEM was purchased from Lily France, Fegersheim, France. Antibodies against NF-κB P65 (cat. no. 8242S), NF-κB p-P65 (ser536) (cat. no. 3033S), Caspase-3 (cat. no. 9662S), Cleaved Caspase-3 (cat. no. 9661S), γ-H2AX (cat. no. 9718S), IKKβ (cat. no. 9936T), p-IKKβ (cat. no. 9936T), Ikb-α (cat. no. 9936T), p-Ikb-α (cat. no. 9936T), and GAPDH (cat. no. 51332S) were purchased from Cell Signaling Technology, USA. Antibody against Histone H3 (cat. no. RM2005) was purchased from Beijing Ray Antibody Biotech, Beijing, China. Antibody against OXCT1 (cat. no. NBP1-82462) was purchased from Novus Biologicals, USA. Annexin V-APC and propidium iodide (PI) were purchased from BioLegend, San Diego, USA.

### Stable Cell Lines

ShRNAs in the PLKO-Puro lentiviral vector against OXCT1 were purchased from Sangon Biotech, Shanghai, China. The coding sequence of OXCT1 was inserted into the pLV-Bsd lentiviral vector. The main plasmid and helper plasmid were packaged into the virus using 293T cells as the host. The target cell lines were then infected using the resulting virus.

### Real−Time Quantitative PCR

RNAiso Plus (TaKaRa, Kusatsu, Japan) was used to extract the total RNA of cells. In accordance with the kit’s instructions (Biomake, Houston, USA), total RNA was subjected to reverse transcription to obtain cDNA. Then, we used 2× SYBR Green qPCR Master Mix (Biomake, Houston, USA) to quantify the target genes. The primer sequences of GAPDH and OXCT1 are as follows:

GAPDH: forward primer: 5ʹ-TGCACCACCAACTGCTTAGC-3ʹ,
reverse primer: 5ʹ- GGCATGGACTGTGGTCATGAG-3ʹ;
OXCT1: forward primer: 5ʹ-GAGAGGAACTTCCCCGCGAT-3ʹ,
reverse primer: 5ʹ-TCCACATAGCCCAAAACCACC-3ʹ.

### Immunohistochemistry

Tumor specimens were fixed in 10% formalin and subsequently embedded in paraffin. Tumor sections were prepared, blocked with 5% BSA for 2 h, and incubated overnight with the primary antibody. They were subsequently incubated for 30 min with the appropriate secondary antibody. Then, the DAB developer was added for further development. Finally, the slides were counterstained with hematoxylin and eosin, and examined under a light microscope (Olympus, Japan).

### Western Blot Analysis

The cells were washed twice with PBS and lysed in RIPA buffer with a proteinase inhibitor cocktail to extract total protein. Nuclear and cytoplasmic components were separated using nuclear and cytoplasmic extraction reagents (Thermo Fisher Scientific, Waltham, USA) according to the manufacturer’s protocol. A total of 20 μg of protein samples was separated on 10% polyacrylamide SDS gels and transferred to polyvinylidene difluoride membranes. The target proteins were detected using western blot analysis.

### Colony Formation Assay

1000 cells were seeded into six-well plates and cultured at 37°C overnight. Then, 50 nM GEM (**Supplementary Figure 1**) was added into the wells. After 12 days, the cells were fixed with 4% paraformaldehyde and then stained with 0.1% crystal violet. Finally, the colonies were counted using a light microscope (Olympus, Japan).

### Apoptosis Assay

The cells were seeded into six-well plates and cultured for 24 h. Then, 50 nM GEM or/and 20 μM BAY 11-7082 were added to the culture for 72 h. Thereafter, the cells were harvested and resuspended using binding buffer. Subsequently, the cells were supplemented with Annexin V-APC and PI. The mixture was incubated at room temperature for 15 min. Finally, the percentage of apoptotic cells was detected using FACSCanto II (BD, USA). All data were analyzed with FlowJo V.10.0.

### Real-Time Cell Analysis

The cell cytotoxicity assay of PDAC cells was performed using an xCELLigence Real-Time Cell Analyzer-Multiple Plate system (Roche Applied Science). This platform was used to measure the viable adhesive target cells in real-time. The cells were resuspended and plated into each well at 37°C with 5% CO_2_. When the normalized cell index reached 1 (about 24 h after plating), the cells were treated with the corresponding agents. The normalized cell index was automatically recorded every 15 min until the end of 72 h (i.e. 48 h after treatment), after which, the final statistical data was recorded. Cell index data for each group were represented as the mean value from three independent wells.

### CCK-8 Assay

For the CCK-8 assay, PDAC cells were plated at the density of 3000 cells/well in the 96-wells plates. After 12 h of culturing, the cells were treated with the gradient concentration of GEM (0.1, 1, 10, 100, 1000, and 10000 nM). Then, the cells treated with GEM were incubated at 37°C in an incubator supplied with 5% CO_2_ for 48 h. Finally, the cell viability was determined using the CCK-8 assay (Biomake, 10μL/well). The cytotoxic activities of PDAC cells were measured under the 450 nm using an immunosorbent instrument (BioTek Synergy H1).

### GSEA

The data of 161 PDAC samples were downloaded from the TCGA database and divided into two groups based on OXCT1 expression level. Pancreatic cancer tissues were subjected to GSEA on the basis of the mRNA expression level of OXCT1 using KEGG gene sets. Then, we selected significantly differentially enriched pathways in accordance with the *P* value threshold and ES. Finally, the absolute value of *P* < 0.05 was considered to represent a statistically significant difference.

### *In Vivo* Tumorigenesis Assay

PDAC cell line MIA PaCa-2 (pLV-vector and pLV-OXCT1) was subcutaneously injected into the BALB/c nude mice. Ten days later, the mice were intraperitoneally injected with saline, GEM (50 mg/kg/week), or a combination of GEM (50 mg/kg/week) and BAY 11-7082 (5 mg/kg/3 days). Tumor sizes were examined every 2 days. The mice were sacrificed after 34 days. All procedures were approved by the Institutional Animal Care and Use Committee of Tianjin Medical University Cancer Institute and Hospital.

### Statistical Analysis

GraphPad Prism 8.0.1 was used for statistical analysis. Each experiment was conducted in triplicate and conducted three or more times. Data were presented as mean from all experiments (± standard error). The Kaplan−Meier method and log-rank test were applied to determine the difference in the survival of patients with PDAC. Single-factor analysis of variance (one-way ANOVA) was used to compare multiple groups. Two-tailed independent-sample Student’s t/tʹ tests was used to compare the two groups. *P* value < 0.05 was considered to be statistically significant.

## Results

### OXCT1 Was Screened as the Key Gene Associated With GEM Resistance in PDAC

**Figure 1:**
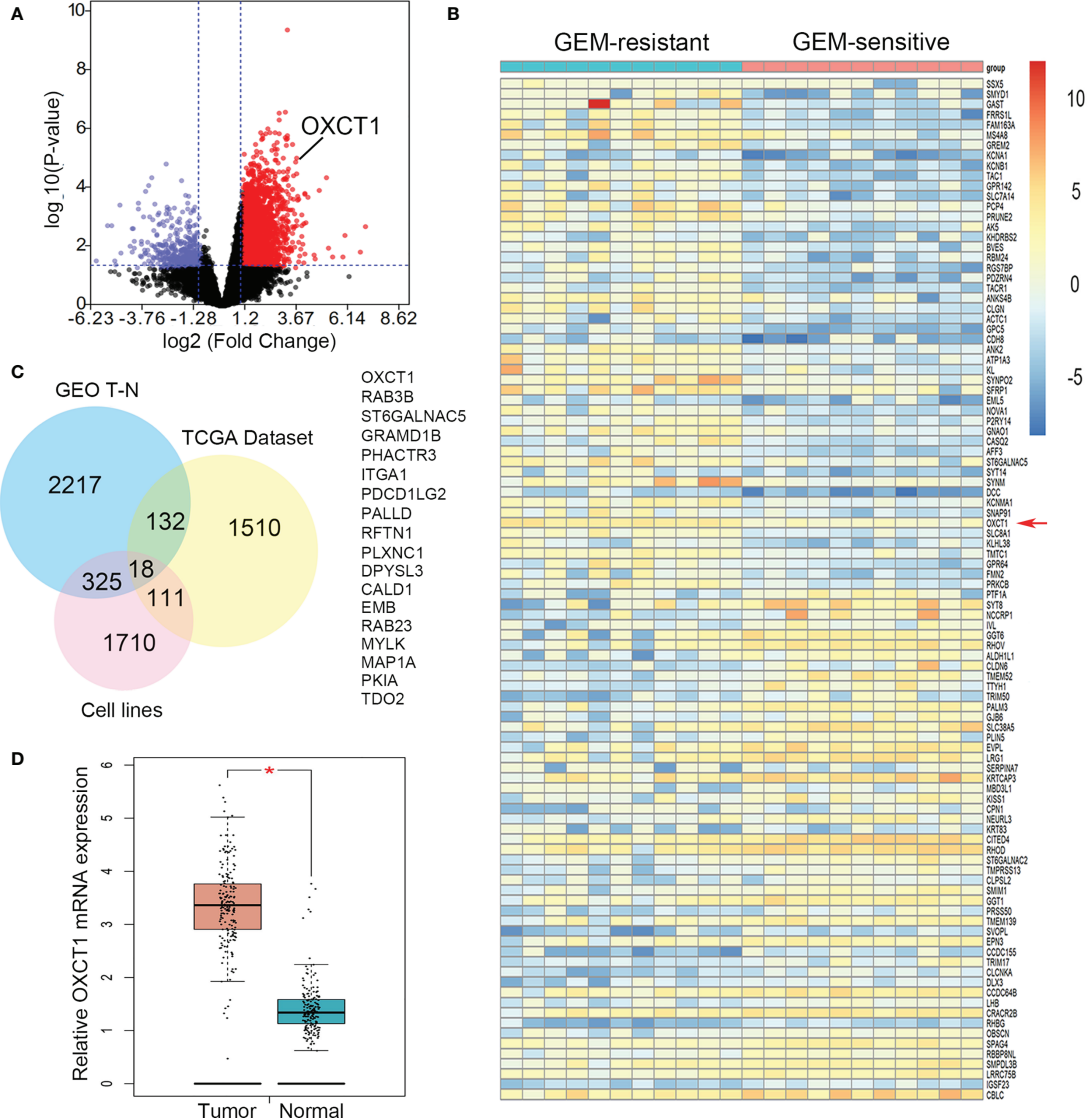
Identification of OXCT1 through DEG analysis. **(A)** Volcano plot depicting the DEGs between GEM-resistant and -sensitive PDAC groups in the TCGA public database. **(B)** Heatmap showing the expression of the top DEGs. **(C)** Venn diagram in which OXCT1 was identified as one of the core intersections of DEGs in the GEO and TCGA datasets. **(D)** Boxplot showing the basic mRNA expression of OXCT1 in tumor and normal tissues in the combined TCGA and GTEx datasets. **P < 0.05.

First, the data on GEM-resistant and -sensitive PDAC groups in the TCGA public database were subjected to differential gene analysis, and the results were represented using a volcano plot (**Figure 1A**). A total of 2955 up-regulated and 471 down-regulated genes with a significant *P* value and FC were identified. Up-regulated genes were marked with red plots, and down-regulated genes with blue plots. The top 100 DEGs between GEM-resistant and -sensitive PDAC groups were presented as a heatmap based on log FC and *P* values (**Figure 1B**). Then, we analyzed multiple datasets containing information on patients with PDAC and differential sensitivity to GEM in TCGA, paired PDAC tumor and normal tissues, and cell lines resistant to GEM in GEO. Finally, 18 critical genes were identified as shown using the Venn diagram in **Figure 1C**. These genes included OXCT1, RAB3B, ST6GALNAC5, GRAMD1B, PHACTR3, ITGA1, PDCD1LG2, PALLD, RFTN1, PLXNC1, DPYSL3, CALD1, EMB, RAB23, MYLK, MAP1A, PKIA, and TDO2 (**Figure 1C**). Moreover, OXCT1 was identified as a valuable and critical gene on the basis of the combined summary rank of *P* values and log FC. Next, we selected all PDAC samples in the TCGA database and normal tissues in the GTEx database as the validated datasets for further validating differential mRNA levels between tumor and normal tissues. The mRNA expression of OXCT1 in tumor tissues was found to be significantly higher than that in normal tissues (**Figure 1D**).

### OXCT1 Was Highly Expressed in PDAC

**Figure 2:**
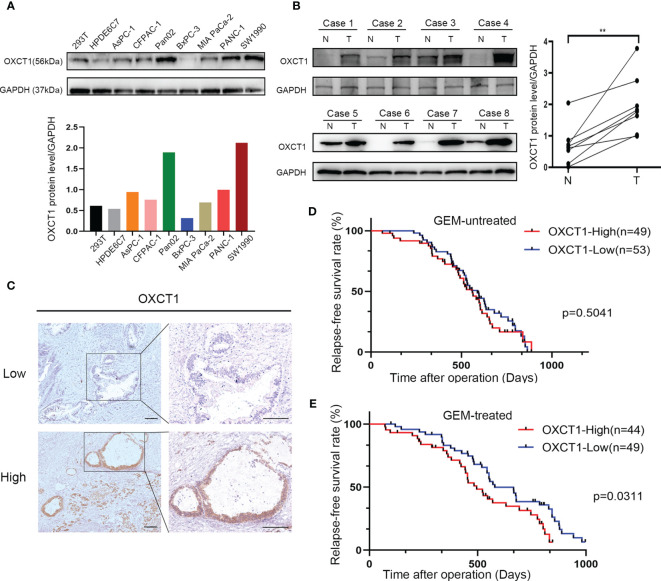
High OXCT1 expression in patients with PDAC treated with GEM predicted poor RFS. **(A)** Basal expression levels of OXCT1 in seven classical pancreatic cancer cell lines, a human pancreatic ductal epithelial cell line (HPDE6C7), and 293T cell line were analyzed through western blot analysis. OXCT1/GAPDH protein expression level is depicted in the histogram. **(B)** Western blot analysis of OXCT1 levels in eight paired PDAC tumorous and adjacent normal pancreatic tissues (T, tumor tissues; N, normal tissues). The corresponding statistics are presented in the line chart. **(C)** Immunohistochemical analysis of OXCT1 protein expression in PDAC specimens. **(D)** Association between tumor OXCT1 expression levels and RFS in 102 patients with PDAC who were not treated with GEM (*P* = 0.5041 based on log-rank test). **(E)** Association between specimen OXCT1 expression levels and RFS in 93 patients with PDAC who were treated with GEM (*P* = 0.0311 based on log-rank test). **P < 0.01.

We performed western blot analysis to verify the expression level of OXCT1 in PDAC and transformed epithelial cell lines, primary tumor tissues, and paired adjacent normal pancreatic tissues. The protein level of OXCT1 in the other six pancreatic cancer cell lines, except for the BxPC-3 cell line, was found to be higher than that in 293T and HPDE6C7 cell lines (**Figure 2A**). Despite interindividual variations, we found that the expression of OXCT1 was significantly higher in cancer tissues than in para-cancerous tissues (**Figure 2B**).

### OXCT1 Was Inversely Associated With RFS in Patients With PDAC Subjected to GEM Treatment

OXCT1 immunohistochemical staining was conducted on the surgical tissue samples of 195 patients with PDAC. The tissue samples were divided into two groups (low and high) based on their H-score (**Figure 2C**). Besides, we divided the patients into GEM-untreated and -treated groups based on the treatment modality. To determine the pathologic significance of OXCT1 expression regarding PDAC progression in GEM-treated group, we evaluated the correlation between OXCT1 expression and established PDAC prognostic factors (**Table 1**). We found that OXCT1 expression was positively correlated with lymph node metastasis and vessel invasion in PDAC specimens (**Table 1**). According to Kaplan–Meier analysis, the RFS of the two groups exhibited significant differences among 93 patients treated with GEM, whereas no significant difference was observed among 102 patients who were not treated with GEM (**Figures 2D, E**). In the GEM-treated group, the patients whose tumors expressed high level of OXCT1 exhibited a significantly shorter RFS than those whose tumors expressed no or low level of OXCT1 (median RFS: 495 days versus 579 days, *P*=0.0311). To elucidate the role of OXCT1 in PDAC progression, we also performed univariate and multivariate analyses of clinical follow-up data for our cohort of PDAC patients (**Table 2**). Consistently, OXCT1 expression was found to be negatively correlated with RFS in both analyses, that is, overexpressed OXCT1 predicted shorter RFS, supporting that OXCT1 could be used as a molecular marker to predict the survival prognosis of PDAC.

**Table 1:** Correlation of OXCT1 expression to clinicopathologic features in PDAC.

Parameters	OXCT1(*n*)		χ^2^	*P*
	Low	High		
Age, years			0.571	0.450
<60	24	25		
≥60	25	19		
Gender			0.610	0.435
Male	24	18		
Female	25	26		
Histologic grade			2.444	0.118
G1,G2	28	18		
G3	21	26		
Tumor size			2.521	0.112
T1	27	17		
T2	22	27		
LN metastasis			3.915	**0.048** ^a^
N0	29	17		
N1	20	27		
Vessel invasion			5.631	**0.018** ^a^
M0	31	17		
M1	18	27		

Statistical data on OXCT1 expression in relation to clinicohistopathologic features for surgical PDAC specimens. P values were calculated using the χ^2^ test.

LN, lymph node.

a Statistically significant (P < 0.05).

**Table 2:** Univariate and multivariate analysis of clinicopathologic factors for RFS.

Characteristics	Univariate Cox			Multivariate Cox		
	Hazard ratio	95% CI	*P*	Hazard ratio	95% CI	*P*
Age (<60 vs. ≥60)	0.785	(0.491, 1.254)	0.311			
Gender (Male vs. Female)	0.979	(0.608, 1.575)	0.929			
Tumor size (T1 vs. T2)	1.491	(0.926, 2.401)	0.100			
Histologic grade (G1,2 vs. G3)	1.662	(1.034, 2.670)	**0.036** ^a^	1.653	(1.028, 2.659)	**0.038** ^a^
LN metastasis (N0 vs. N1)	1.435	(0.895, 2.300)	0.133			
Vessel invasion (No vs. Yes)	1.081	(0.674, 1.734)	0.746			
OXCT1(Low vs. High)	1.711	(1.043, 2.807)	**0.034** ^a^	1.703	(1.035, 2.802)	**0.036** ^a^

Univariate analysis: log rank; multivariate Cox proportional hazards analysis.

LN, lymph node.

a Statistically significant (P < 0.05).

### Changes in OXCT1 Expression Level Affected GEM Resistance Capability of PDAC

**Figure 3:**
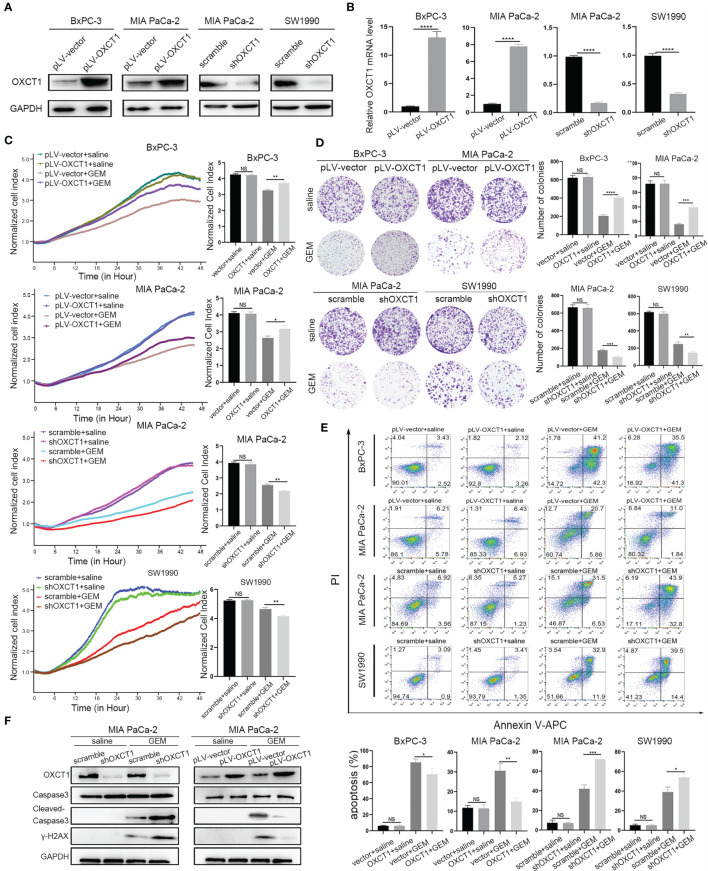
Effect of OXCT1 on GEM resistance in PDAC cells. **(A)** Western blot analysis of proteins extracted from the two OXCT1-overexpressing stable cell lines (BxPC-3 and MIA PaCa-2) and two OXCT1-knockdown cell lines (MIA PaCa-2 and SW1990). **(B)** Real−time quantitative PCR analysis of OXCT1 expression levels in PDAC stable cell line included in **(A)**. **(C)** OXCT1-overexpressing stable cell lines (BxPC-3 and MIA PaCa-2) and OXCT1-knockdown cell lines (MIA PaCa-2 and SW1990) were treated with either 50 nM GEM or saline before conducting the cytotoxicity assay. Real-time cell index measurements (xCELLigence) of the live target cells cultured with GEM or saline are shown in **(C)**. The corresponding 72 h normalized cell index is shown as the histogram**. (D)** Representative images and quantification using the colony formation assay of the indicated cell lines that were treated for 72 h with GEM or saline. **(E)** Flow cytometry was performed to measure the apoptosis rates of the indicated cell lines treated with 50 nM GEM or saline for 72 h The corresponding statistics are presented in the histogram. **(F)** Detection of cleaved-caspas3 and γ-H2AX in OXCT1 overexpression and knockdown MIA PaCa-2 cell lines. The data are expressed as mean ± SEM from three independent experiments. *P < 0.05; **P < 0.01; ***P < 0.001; ****P < 0.0001 (one-way ANOVA). NS, not significant.

We first constructed stable cell lines to detect GEM resistance capability of PDAC cells on the basis of the basic expression level of OXCT1. We transfected pLV-OXCT1 lentivirus into BxPC-3 and MIA PaCa-2 cell lines to increase OXCT1 expression and constructed sequence-specific shRNA target OXCT1 to silence the expression of OXCT1 in the MIA PaCa-2 and SW1990 cell lines. The expression levels of OXCT1 protein and mRNA in the pLV-OXCT1 group were significantly higher than those in the pLV-vector group. Correspondingly, the expression levels of OXCT1 protein and mRNA in the shRNA-OXCT1 group were significantly lower than those in the scramble group (**Figures 3A, B**). Antiapoptosis is one of the main mechanisms of GEM resistance. First, to investigate a suitable GEM concentration for the following experiments, we observed the GEM cytotoxic effect in 3 PDAC cell lines, including BxPC-3, MIA PaCa-2, SW1990 cells (**Supplementary Figure 1**). Based on the 3 PDAC cells’ IC50 value, we confirmed that 50 nM was the most suitable concentration for cell lines treatment. Next, we assessed the cytotoxic potential of GEM (50 nM) using real-time cell index (xCELLigence) measurements. In contrast to OXCT1 silencing, OXCT1 overexpression could significantly improve the capability of PDAC to tolerate GEM cytotoxicity (**Figure 3C**). Additionally, under GEM treatment (50 nM), OXCT1-overexpressing cells formed more colonies than OXCT1-silenced cells (**Figure 3D**). Moreover, apoptosis flow cytometry assays suggested that the upregulation of OXCT1 decreased the apoptosis rate of PDAC cells induced by GEM (50 nM). However, the downregulation of OXCT1 showed the opposite effect (**Figure 3E**). Overall, our data suggested that OXCT1 could significantly improve the resistance of PDAC cells to GEM.

Further, we confirmed the effect of OXCT1-induced chemoresistance of GEM. Cleaved‐caspase3 and γ-H2AX were tested using western blot analysis (**Figure 3F**). Compared with the control group, cleaved-caspase3 expression was found to be increased in the sh-OXCT1 group, and decreased in the pLV-OXCT1 group when treated with 50 nM GEM. Similarly, γ-H2AX expression exhibited the same trend. These results reflect that OXCT1 overexpression increases the PDAC cells’ refractory capability to DNA damaging effect induced by GEM and OXCT1 knockdown enhances the PDAC cells’ sensitivity to this chemotherapy.

### OXCT1 Induced GEM Resistance Through NF-κB Signaling Pathway

**Figure 4:**
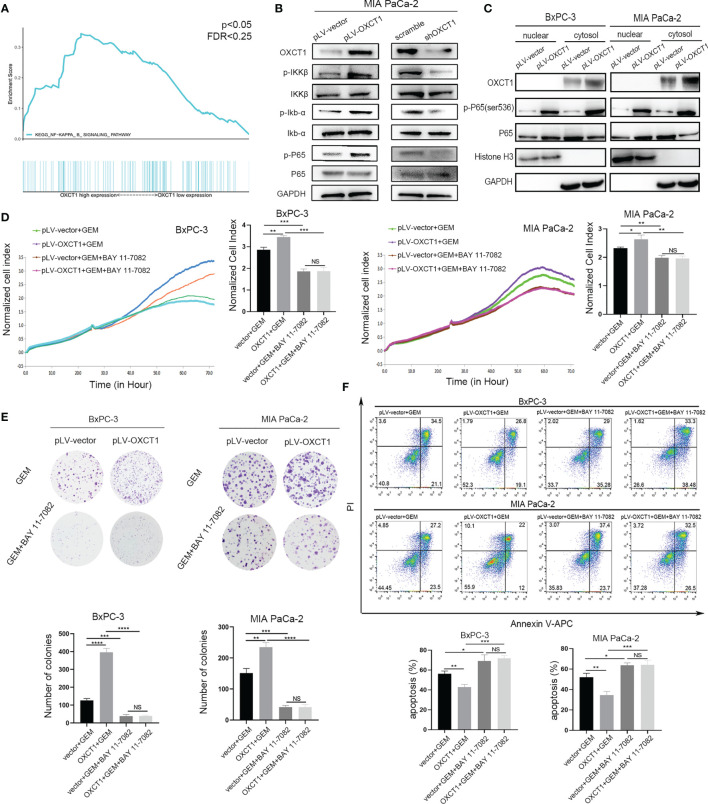
OXCT1 promoted GEM resistance through the NF-κB signaling pathway. **(A)** The TCGA dataset was subjected to GSEA on the basis of OXCT1 expression. **(B)** The expression levels of p-IKKβ, p-Ikb-α, and p-P65 in MIA PaCa-2 cell lines with OXCT1 overexpression and knockdown were detected using western blot analysis. **(C)** Western blot analysis of the fractionated BxPC-3 and MIA PaCa-2 cells stably transfected with the empty vector or OXCT1. **(D–F)** BxPC-3 and MIA PaCa-2 cell lines with or without OXCT1 overexpression were treated with GEM or the combination of GEM and BAY 11-7082. Cell cytotoxicity was analyzed using RTCA **(D)**. Cell cloning capability was analyzed using the colony formation assay **(E)**. Apoptosis was detected *via* flow cytometry **(F)**. The data are expressed as mean ± SEM from three independent experiments. *P < 0.05; **P < 0.01; ***P < 0.001; ****P < 0.0001 (one-way ANOVA). NS, not significant.

We validated the phenotype of OXCT1 through the above experiments. GSEA was conducted on the basis of PDAC samples in the TCGA database to identify the concrete downstream mechanism of OXCT1’s phenotypic effect. We found that the NF-κB signaling pathway was remarkably positively correlated with OXCT1 expression with a statistically significant *P* value and fold change (*P*<0.05, FDR<0.25) in accordance with the mRNA expression of OXCT1 (**Figure 4A**). To determine the key role of the NF-κB signaling pathway in the OXCT1-induced chemoresistance, we tested the expression of IKKβ, Ikb-α, and P65 and their phosphorylation levels in the NF-κB signaling pathway. As is shown in the western blot analysis (**Figure 4B** and **Supplementary Figure 3**), upregulation of OXCT1 led to an increase in the phosphorylation levels of IKKβ, Ikb-α, and P65. Conversely, the knockdown of OXCT1 decreased the levels of phosphorylation of IKKβ, Ikb-α, and P65. The above results indicate that OXCT1 promotes the activation of the NF-κB signaling pathway. Besides, in this study, NF-κB activation was caused by OXCT1 upregulation, and not by GEM action. We also examined the nuclear localization of P65 protein by subjecting fractionated proteins from PDAC cell lines with or without OXCT1 overexpression to western blot analysis to validate GSEA results. The levels of the activated nuclear-form p-P65 were significantly higher in pLV-OXCT1 cells than in pLV-vector cells (**Figure 4C**). BAY 11-7082, an inhibitor of the NF-κB signaling pathway, was used to explore the effects of NF-κB inhibition in the OXCT1-mediated GEM refractoriness. First, we utilized BAY 11-7082 individually to treat BxPC-3 and MIA PaCa-2 cell lines. After 48 h of treatment, we did not observe any difference between the vector and OXCT1-overexpression groups (**Supplementary Figure 4**). Next, as shown in **Figures 4D**–**F**, we conducted real-time cell cytotoxicity, colony, and apoptosis assays with GEM and/or BAY 11-7082 to further validate whether OXCT1 induced chemotherapy resistance in PDAC is *via* the NF-κB signaling pathway. Remarkably, the effect induced by OXCT1 could be mostly abrogated by BAY 11-7082, indicating that the induction of GEM resistance by OXCT1 was dependent on the NF-κB signaling pathway.

### Targeting NF-κB Signaling Pathway Reversed OXCT1-Induced PDAC Resistance to GEM in the Mouse Model

**Figure 5:**
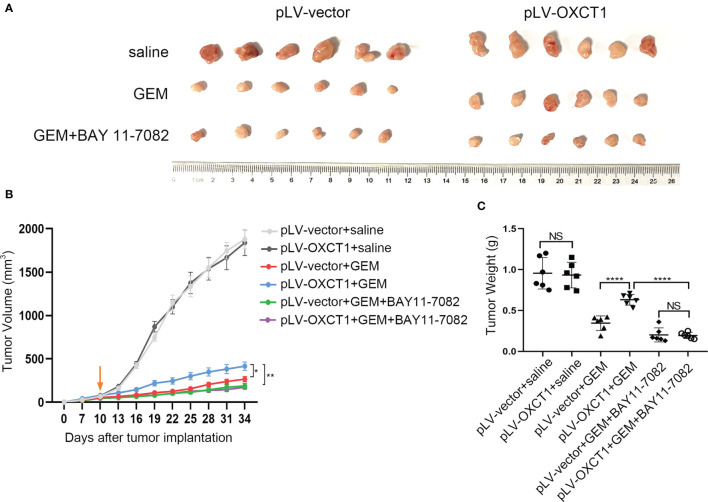
NF-κB inhibitor reversed OXCT1-induced PDAC resistance to GEM in mouse models. **(A–C)** The indicated tumor cells were subcutaneously transplanted into the nude mouse model to develop tumors (n=6 for each group). Ten days later, the mice were treated with saline, GEM, or the combination of GEM and BAY 11-7082. Tumor volumes were measured every 3 days using the calipers. Then, the mice were sacrificed, and tumors were excised. The representative images of the tumors are depicted **(A)**. Repeated measure two-way ANOVA (time × tumor volume) analysis was performed to compare the tumor growth curve among the six groups **(B)**. Tumor weights at the end of the experiment are also shown and were analyzed **(C)**. Data are presented as mean ± SD; *P < 0.05; **P < 0.01; ****P < 0.0001. NS, not significant.

Given that OXCT1 plays an important role in PDAC resistance to GEM *via* the NF-κB signaling pathway, NF-κB targeted therapy may contribute to the effect of chemotherapy on PDAC with high OXCT1 expression. Therefore, subcutaneous mouse xenograft models were used to assess whether the NF-κB signaling pathway inhibitor could enhance the chemotherapy effect upon OXCT1 overexpression. MIA PaCa-2-vector cells and MIA PaCa2-OXCT1 cells were injected into nude mice. BAY 11-7082 was used to block NF-κB pathway in tumor cells. The mice were treated with saline, GEM, or a combination of GEM and BAY 11-7082 to compare the efficacy of GEM with or without targeted NF-κB therapy in MIA PaCa2-vector and MIA PaCa-2-OXCT1 tumors. Compared with that under GEM monotherapy, tumor growth in MIA PaCa-2-OXCT1 tumors was significantly inhibited under GEM + BAY 11-7082 therapy. However, no significant difference was observed in the GEM + BAY 11-7082 group (**Figures 5A, B**). Furthermore, tumor weight demonstrated the same effect (**Figure 5C**). Our data suggest that targeting the NF-κB signaling pathway might be a potential treatment strategy for inhibiting OXCT1-induced PDAC resistance to GEM. Moreover, NF-κB targeted therapy, as a complement to GEM chemotherapy, might offer an alternate option for treating PDAC with high OXCT1 levels.

## Discussion

GEM has been used as the front-line treatment option for PDAC for nearly 24 years (25) despite its extremely limited effects on the survival of patients. The new first-line treatment options including GEM+nab-paclitaxel and FOLFIRINOX (5-FU, leucovorin, irinotecan, and oxaliplatin) have been established during the last few years (13, 16, 26, 27). However, the side effects of FOLFIRINOX limit their effectiveness (28, 29). Therefore, GEM remains an important chemotherapeutic agent in PDAC therapy. PDAC is the most malignant tumor with limited therapeutic options for patients who often present resistance to GEM (15, 30). Therefore, finding an efficient therapeutic target to inhibit GEM resistance remains a great challenge, but it is essential to improve the survival of patients. Elucidating the molecular mechanisms underlying the chemoresistance to GEM is extremely valuable.

In this study, we used public databases to analyze PDAC chemoresistance, and then, selected the screened intersection gene OXCT1 as the target for inhibiting PDAC resistance to GEM. Ketolysis is an avenue for cells to obtain energy when hungry or stressed (31). OXCT1 is a key enzyme in ketone body metabolism that catalyzes the first and rate-determining step of ketolysis (12, 24, 32–34). It promotes ketone metabolism, thereby increasing ATP production.

PDAC tumor tissue fibrosis is a serious complication, and the tumor microenvironment of PDAC is characterized by the lack of oxygen and blood supply, which further promotes malnutrition (19, 35–37). Therefore, in PDAC, the metabolic model may change to a certain extent, and ketolysis is enhanced (35, 38, 39). In 2016, De Huang et al. found that OXCT1 is activated in liver cancer cells and facilitates ketone body utilization as an energy source for cell survival and growth under malnutrition (23). Their findings provided a new method for treating patients with HCC through regulating OXCT1, a key enzyme of ketolysis. Recently, Ozsvari et al. demonstrated that a ketone shuttle is present in tumors (40). They found that some cancer-associated fibroblasts can produce ketone bodies, which are then taken up and utilized by the surrounding tumor cells. These evidences (41–45) prove that OXCT1 can help drive tumor progression and metastasis, and also confirm the reliability of our screening results.

In our study, we first evaluated the association between OXCT1 expression and RFS in patients with PDAC who were treated with GEM and found a significant negative correlation between high OXCT1 expression and the patients’ RFS (**Figure 2E**). Subsequently, the validation was performed at the cell-line level *via* the overexpression and knockdown of OXCT1 in PDAC cell lines (BxPC-3, MIA PaCa-2, and SW1990). We found that GEM-treated cell lines significantly outperformed the cell line in the control group in terms of antiapoptotic effect following OXCT1 overexpression (**Figures 3C**–**F** and **Supplementary Figures 2A**–**C**). Conversely, the antiapoptotic capability of cell lines were significantly reduced after OXCT1 knockdown under the same treatment conditions. OXCT1 was identified as a bona fide metabolic oncogene, which promotes the occurrence and development of tumors in 2012 (44, 46). Our conclusion is consistent with theirs. Next, we questioned how OXCT1 caused GEM chemoresistance in PDAC cells.

Through GSEA, we discovered that the NF-κB signaling pathway was significantly positively correlated with OXCT1 expression. Therefore, we postulated that the NF-κB signaling pathway might be downstream of OXCT1 (**Figure 4A**). NF-κB is a transcription factor that is regulated by many stimuli, including chemotherapeutic drugs, hypoxia, and malnutrition, and some cytokines have recently emerged as popular targets for cancer research (47). In BxPC-3 and MIA PaCa-2 cell lines, the activated nuclear form p-P65 was found to be significantly elevated when OXCT1 was overexpressed (**Figure 4C**). Previous research revealed that when NF-κB is activated, it translocates into the nucleus where it binds to the cognate sequences in the promoter region of multiple genes that encode factors in tumor promotion and proliferation, cytokines, antiapoptotic proteins, and genes related to chemoresistance (48–50). The results of our study demonstrated that the resistance to GEM induced by OXCT1 overexpression was significantly inhibited upon blocking the NF-κB signaling pathway using an inhibitor (**Figures 4D**–**F**). Additionally, we reached the same conclusion through *in vivo* validation through conducting a tumor formation assay in nude mice. The above evidence suggested that OXCT1 promotes PDAC resistance to GEM *via* the NF-κB signaling pathway.

**Figure 6:**
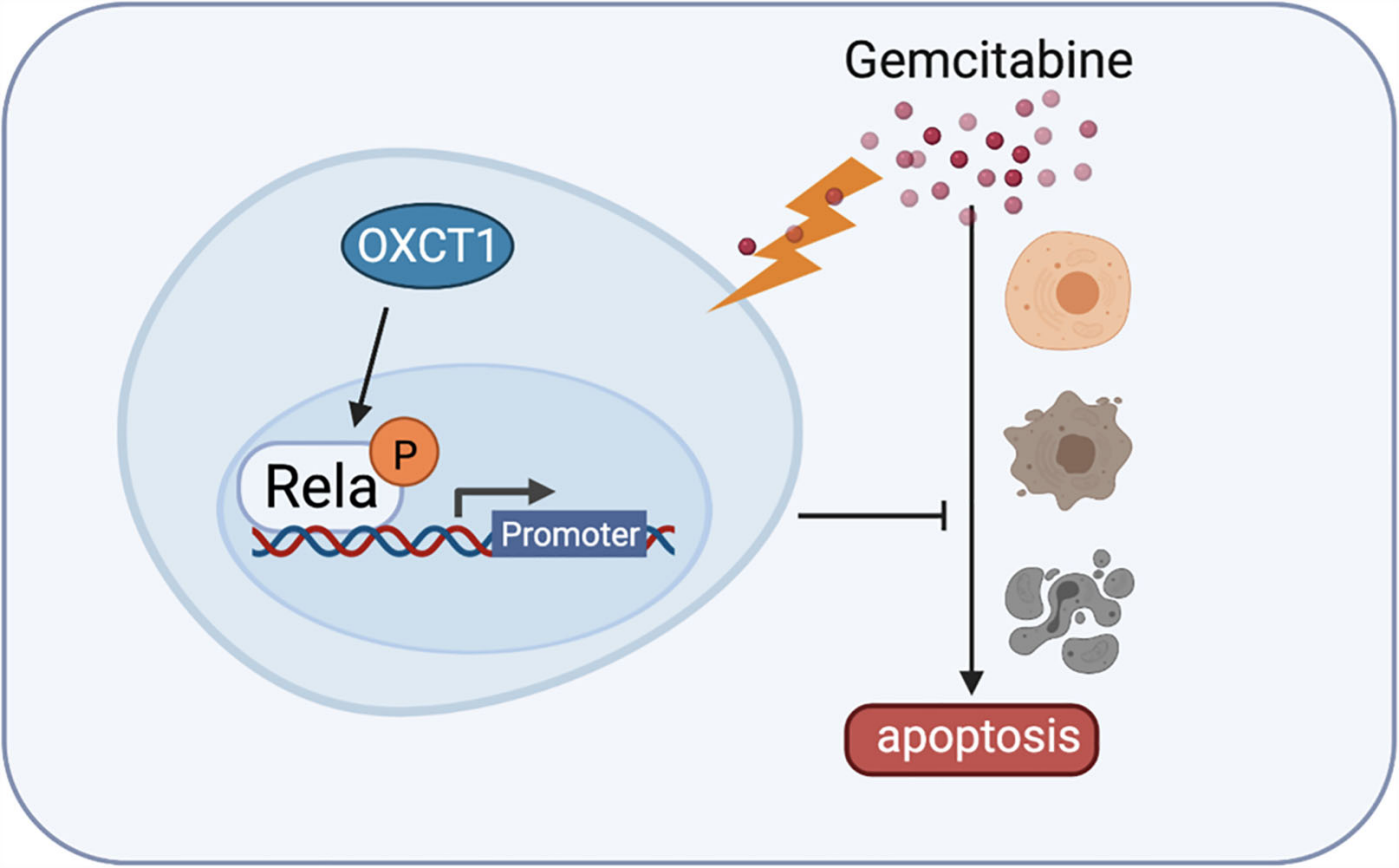
Detailed mechanism of gemcitabine resistance in PDAC induced by OXCT1. Schematic diagram shows that OXCT1 can significantly enhance the resistance to gemcitabine by activating the NF-κB signaling pathway in PDAC cells, thereby inhibit cell apoptosis, and finally promote the occurrence and progression of PDAC.

Upon validation using cell lines and mouse model (**Figure 5**) experiments, we confirmed that OXCT1 significantly promotes the resistance of PDAC cells to the cytotoxic effect of GEM *via* the NF-κB signaling pathway (**Figure 6**). Our results indicate that OXCT1 is a potential chemoresistance target and a crucial biomarker for assessing the prognosis and determining the optimal chemotherapy strategies for patients with PDAC. Through our study, we identified OXCT1 as a new research target, thereby providing a useful reference for PDAC precision therapy.

## Data Availability Statement

The datasets presented in this study can be found in online repositories. The names of the repository/repositories and accession number(s) can be found in the article/**Supplementary Material**.

## Ethics Statement

The studies involving human participants were reviewed and approved by Ethics Committee of Tianjin Medical University in Tianjin Medical University Cancer Institute and Hospital. The patients/participants provided their written informed consent to participate in this study. The animal study was reviewed and approved by Ethics Committee of Tianjin Medical University in Tianjin Medical University Cancer Institute and Hospital. Written informed consent was obtained from the individual(s) for the publication of any potentially identifiable images or data included in this article.

## Author Contributions

JD, HL, YL, LB, HW, and CG: design and initiation of the study, quality control of data, data analysis and interpretation, and manuscript preparation and editing. YX: perform the bioinformatics data analysis. JY, HS, DX, and YZ: data acquisition. All authors contributed to the article and approved the submitted version.

## Funding

This work was supported by the National Natural Science Foundation of China (grants 82030092, 81720108028, 82072657, 81802432, 82072716, 81802433, 82072659, 81871968, and 81871978), Key Program of Prevention and Treatment of Chronic Diseases of Tianjin (17ZXMFSY00010), the programs of Tianjin Prominent Talents, Tianjin Eminent Scholars, Tianjin Natural Science Foundation (18JCJQJC47800, 19JCJQJC63100, 19JCYBJC26200, and 20JCQNJC01330), Tianjin Postgraduate Research and Innovation Project(2019YJSB104), and Tianjin Research Innovation Project for Postgraduate Students(2019YJSB104).

## Conflict of Interest

The authors declare that the research was conducted in the absence of any commercial or financial relationships that could be construed as a potential conflict of interest.

## Publisher’s Note

All claims expressed in this article are solely those of the authors and do not necessarily represent those of their affiliated organizations, or those of the publisher, the editors and the reviewers. Any product that may be evaluated in this article, or claim that may be made by its manufacturer, is not guaranteed or endorsed by the publisher.

## Supplementary Material

Supplementary Figure 1 Determination of gemcitabine IC50 in the *in vitro* study. CCK-8 (cell counting kit-8) was used to analysis IC50 of BxPC-3, MIA PaCa-2 and SW1990 cell lines. The data are expressed as mean ± SEM from three independent experiments.

Supplementary Figure 2 OXCT1-knockdown BxPC-3 stable cell line and OXCT1-overexpressing SW1990 stable cell line construction and functional verification. **(A)** Western blot analysis of proteins extracted from OXCT1-knockdown cell line (BxPC-3) and OXCT1-overexpressing cell line (SW1990). **(B)** Representative images and quantification using the colony formation assay of the indicated cell lines that were treated for 72 h with 50 nM GEM or saline. **(C)** Flow cytometry was performed to measure the apoptosis rates of the indicated cell lines treated with 50 nM GEM or saline for 72 h. The corresponding statistics are presented in the histogram. The data are expressed as mean ± SEM from three independent experiments. **P* < 0.05; ***P* < 0.01; ****P* < 0.001; *****P* < 0.0001 (one-way ANOVA).

Supplementary Figure 3 Effect of OXCT1 on NF-κB signaling pathway. Western blot analysis was used to detect the expression levels of p-IKKβ, p-Ikb-α and p-P65 in OXCT1-overexpressing and OXCT1-knockdown MIA PaCa-2 cell lines treated with 50 nM gemcitabine.

Supplementary Figure 4 Effect of NF-κB signaling pathway inhibitor BAY 11-7082 on cell lines apoptosis. **(A)** Flow cytometry was performed to measure the apoptosis rates of OXCT1-overexpressing cell lines (BxPC-3 and MIA PaCa-2) treated with 50 nM GEM or saline for 72 h. The corresponding statistics are presented in the histogram. **(B)** Flow cytometry was performed to measure the apoptosis rates of OXCT1-knockdown cell lines (SW1990 and MIA PaCa-2) treated with 50 nM GEM or saline for 72 h. The corresponding statistics are presented in the histogram. The data are expressed as the means ± SEM from three independent experiments. **P* < 0.05; ***P* < 0.01; ****P* < 0.001; *****P* < 0.0001 (one-way ANOVA).
