# Supplementary figures and images for: OXCT1 Enhances Gemcitabine Resistance Through NF-κB Pathway in Pancreatic Ductal Adenocarcinoma
Source: Front Oncol. 2021 Nov 5;11:698302. doi: 10.3389/fonc.2021.698302 (PMC8602561; doi:10.3389/fonc.2021.698302)

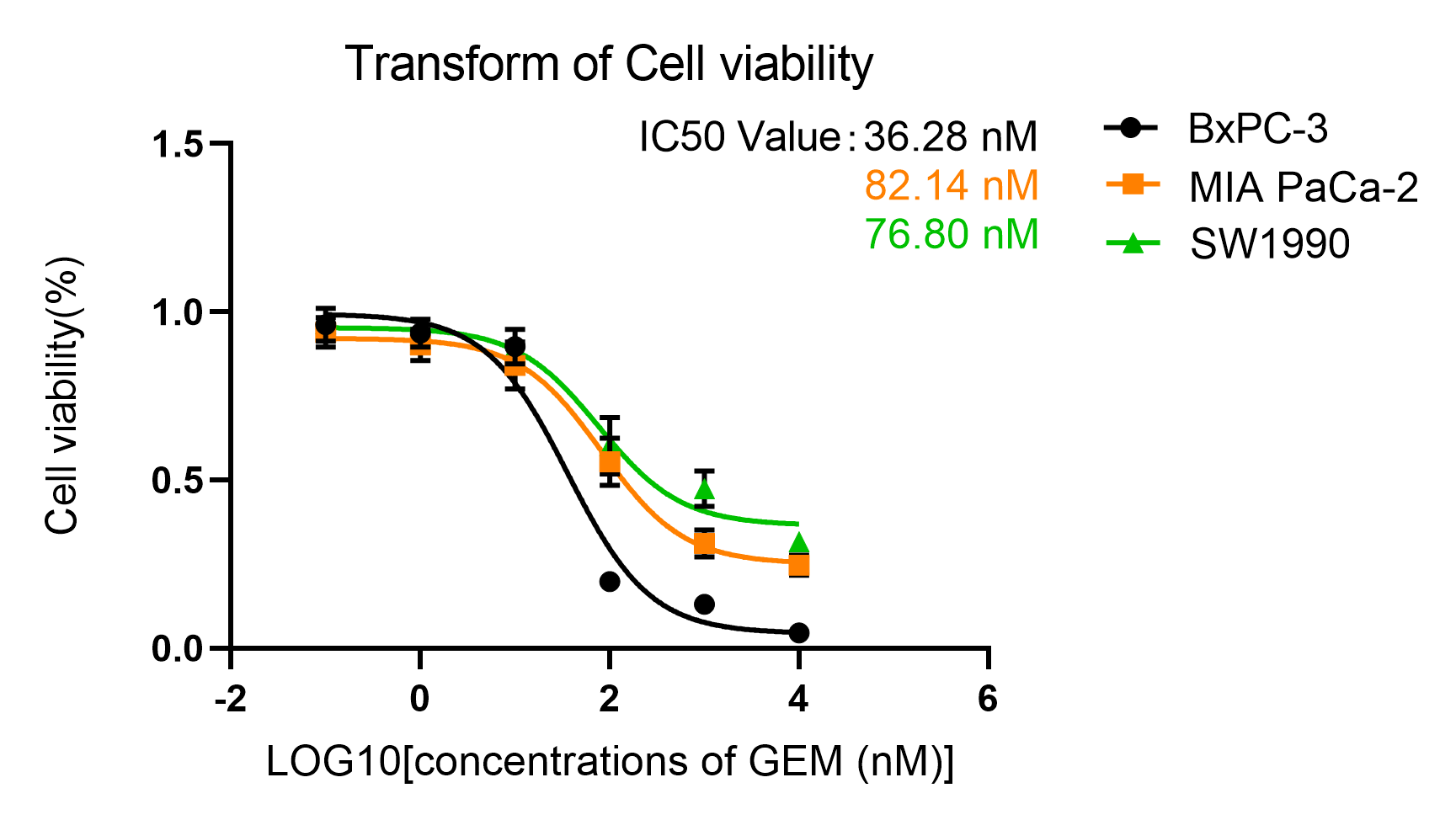

Supplement: Supplementary Figure 1 — Determination of gemcitabine IC50 in the in vitro study. CCK-8 (cell counting kit-8) was used to analysis IC50 of BxPC-3, MIA PaCa-2 and SW1990 cell lines. The data are expressed as mean ± SEM from three independent experiments. [file Image_1.tif]

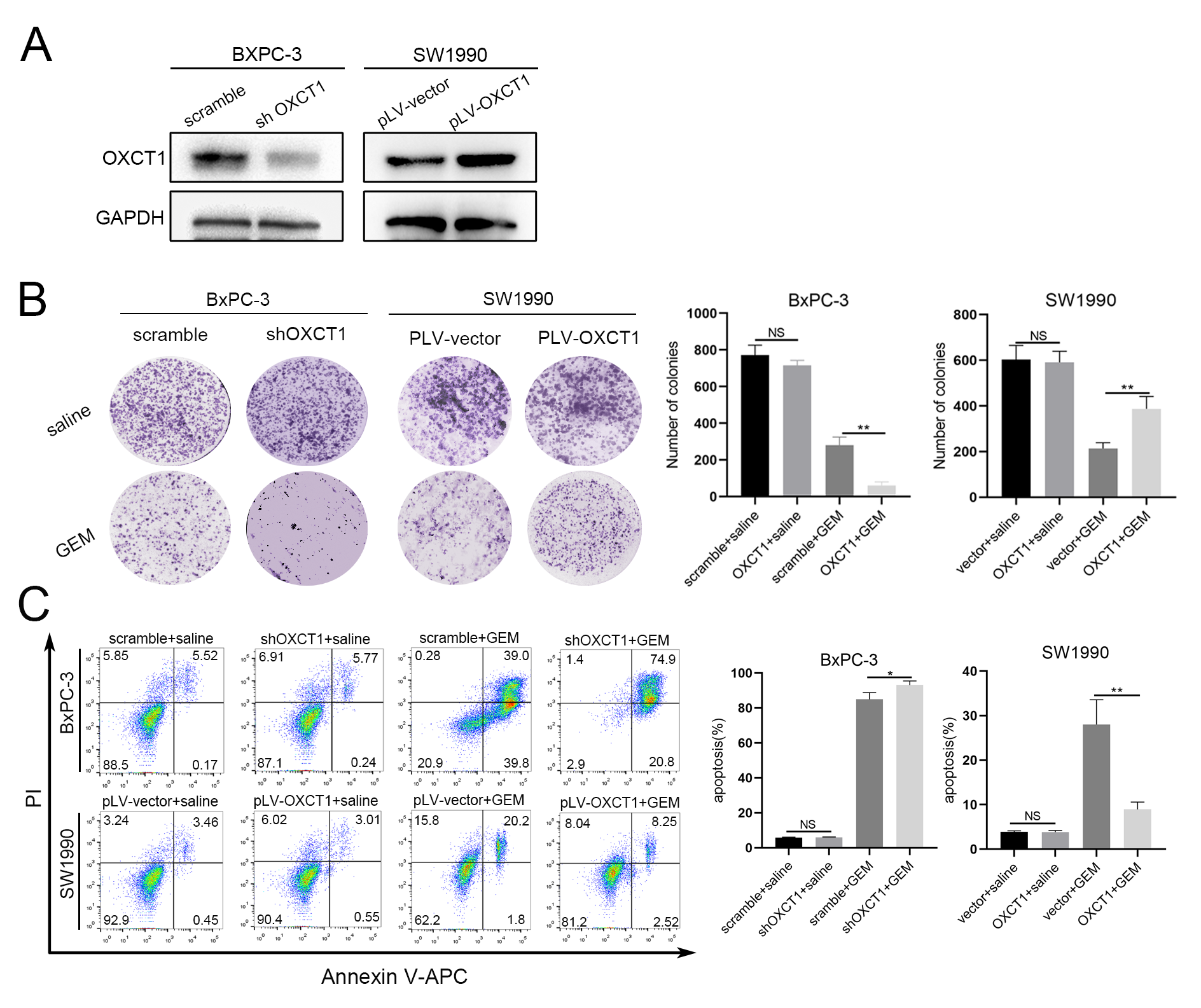

Supplement: Supplementary Figure 2 — OXCT1-knockdown BxPC-3 stable cell line and OXCT1-overexpressing SW1990 stable cell line construction and functional verification. (A) Western blot analysis of proteins extracted from OXCT1-knockdown cell line (BxPC-3) and OXCT1-overexpressing cell line (SW1990). (B) Representative images and quantification using the colony formation assay of the indicated cell lines that were treated for 72 h with 50 nM GEM or saline. (C) Flow cytometry was performed to measure the apoptosis rates of the indicated cell lines treated with 50 nM GEM or saline for 72 h. The corresponding statistics are presented in the histogram. The data are expressed as mean ± SEM from three independent experiments. *P < 0.05; **P < 0.01; ***P < 0.001; ****P < 0.0001 (one-way ANOVA). [file Image_2.tif]

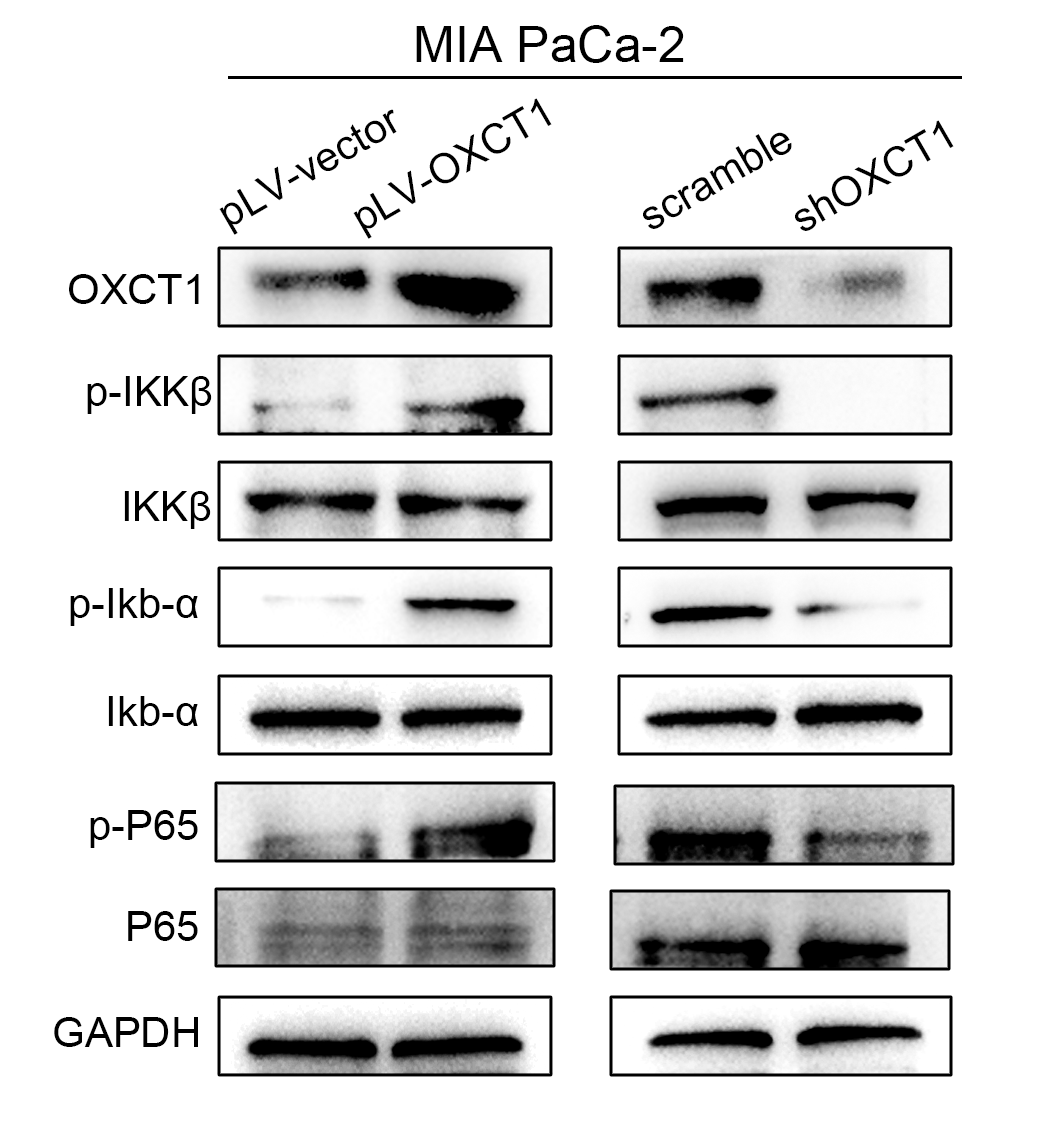

Supplement: Supplementary Figure 3 — Effect of OXCT1 on NF-κB signaling pathway. Western blot analysis was used to detect the expression levels of p-IKKβ, p-Ikb-α and p-P65 in OXCT1-overexpressing and OXCT1-knockdown MIA PaCa-2 cell lines treated with 50 nM gemcitabine. [file Image_3.tif]

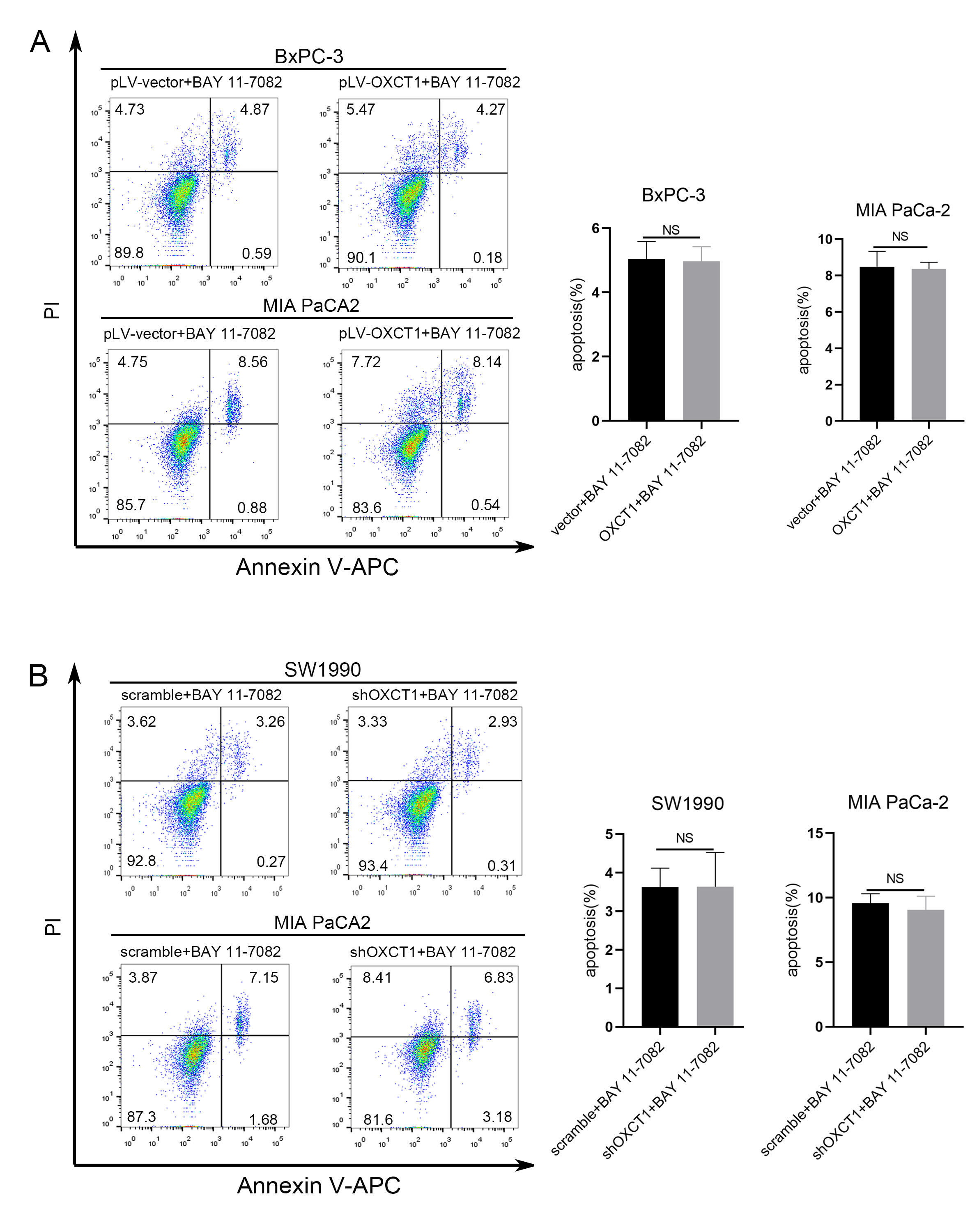

Supplement: Supplementary Figure 4 — Effect of NF-κB signaling pathway inhibitor BAY 11-7082 on cell lines apoptosis. (A) Flow cytometry was performed to measure the apoptosis rates of OXCT1-overexpressing cell lines (BxPC-3 and MIA PaCa-2) treated with 50 nM GEM or saline for 72 h. The corresponding statistics are presented in the histogram. (B) Flow cytometry was performed to measure the apoptosis rates of OXCT1-knockdown cell lines (SW1990 and MIA PaCa-2) treated with 50 nM GEM or saline for 72 h. The corresponding statistics are presented in the histogram. The data are expressed as the means ± SEM from three independent experiments. *P < 0.05; **P < 0.01; ***P < 0.001; ****P < 0.0001 (one-way ANOVA). [file Image_4.tif]
